# *miR4ASD*: A Database of Human microRNAs Associated with Autism Spectrum Disorder

**DOI:** 10.3390/cimb48101044

**Published:** 2026-10-09

**Authors:** Ana Rita Marques, Hugo Martiniano, Joana Vilela, Astrid Moura Vicente

**Affiliations:** 1 Departamento de Promoção da Saúde e Doenças Não Transmissíveis, Instituto Nacional de Saúde Doutor Ricardo Jorge, Avenida Padre Cruz, 1649-016 Lisboa, Portugal; a.rita.marques@insa.min-saude.pt (A.R.M.); hugo.martiniano@insa.min-saude.pt (H.M.); joana.vilela@insa.min-saude.pt (J.V.); 2 BioISI-Biosystems & Integrative Sciences Institute, Faculty of Sciences, University of Lisboa, Campo Grande, C8, 1749-016 Lisboa, Portugal

**Keywords:** ASD, autism, miRNA, microRNA, human, open-source database, *miR4ASD*

## Abstract

Autism spectrum disorder (ASD) is a complex neurodevelopmental condition characterized by substantial etiological and clinical heterogeneity. MicroRNAs (miRNAs) are post-transcriptional regulators of gene expression that play critical roles in brain development. As such, a growing body of work suggests a role of miRNA dysregulation in several human pathologies of the brain, including ASD. However, evidence on miRNA involvement in ASD is dispersed across the literature, limiting its utility for biomarker discovery and functional insights. Here, we present *miR4ASD*, a manually curated and open-access database of human miRNAs implicated in ASD, covering data from case–control studies of both gene expression and genetic variant analysis. This resource integrates structured data from peer-reviewed publications, including study methodology, tissue type, expression alteration (upregulated or downregulated), sample size, number of supporting studies, and reported genetic variants within miRNA genes. To ensure objectivity and precision, 59 studies meeting the strict inclusion criteria were included. *miR4ASD* also integrates miRNA–target interaction data from external resources and allows users to perform functional enrichment analysis of target genes. By providing a dynamic and accessible platform, *miR4ASD* enables the systematic exploration of miRNA involvement in ASD and supports research in diagnostics, biomarker discovery, and systems biology. The database is publicly available on GitHub.

## 1. Introduction

Autism spectrum disorder (ASD) is a heritable neurodevelopmental disorder characterized by impairments in social communication and interactions, along with restrictive and repetitive behaviors [1,2]. Despite extensive genomic research data, the biological mechanisms underlying ASD remain incompletely understood, largely due to the disorder’s high phenotypic and genetic heterogeneity. While protein-coding mutations and structural variants have received considerable attention, non-coding RNAs, particularly microRNAs (miRNAs), are gaining recognition for their roles in neural development and neuropsychiatric conditions. MiRNAs are a class of small, non-coding RNAs that regulate gene expression by promoting mRNA degradation and/or translational repression [3]. They are estimated to regulate more than 60% of the human transcriptome, thereby contributing to fundamental biological processes, including neuronal differentiation, synapse formation, neurogenesis, and immune regulation [4,5].

Over the past decade, the interest in the role of miRNAs as potential biomarkers for ASD has intensified. Multiple studies have implicated specific miRNAs, reporting altered expression patterns in both central (e.g., post-mortem brain tissue) and peripheral (e.g., blood, saliva) biological samples [6,7]. However, these findings remain scattered across independent studies and differ in experimental design and methodology, highlighting the need for integration into a centralized resource with standardized miRNA nomenclature. Although narrative, scoping, and systematic reviews have provided valuable syntheses of the field [8,9,10], they are inherently static and quickly become outdated, as they cannot be continuously updated with emerging evidence.

In contrast, existing databases on miRNA-disease associations, such as the Human MicroRNA Disease Database (HMDD) [11], are very broad in scope and lack some ASD studies. Therefore, it is difficult to compare results across experiments or capture the full range of reported interconnections between a given miRNA and ASD. This limitation emphasizes the need for a specialized resource containing curated data from ASD–control studies, with standardized nomenclature based on the latest release of miRBase, the reference repository for miRNA sequences and annotation [12]. Such a resource can provide a dynamic and continuously evolving platform that integrates both expression studies and genetic variant data, offering a more comprehensive and up-to-date perspective of the contribution of miRNAs to ASD.

To address these gaps, we developed *miR4ASD*, a manually curated database designed to organize and integrate the available evidence on human miRNAs associated with ASD. Based on a comprehensive review of the literature, *miR4ASD* consolidates data from miRNA expression and genetic studies into a centralized and standardized resource, facilitating the identification of consistently reported miRNAs and highlighting areas where evidence remains limited. The database also integrates complementary information from external resources, including miRNA–target interactions, evidence supporting the involvement of target genes in ASD risk, and functional enrichment analysis of target genes. By making these data readily accessible for comparison and further investigation, *miR4ASD* offers a centralized resource to support future research in ASD diagnostics, biomarker discovery, therapeutics, and systems biology.

## 2. Materials and Methods

### 2.1. Strategy for Study Identification

The first step was to search for studies in PubMed, Scopus and Web of Science databases using keyword combinations related to “Autism Spectrum Disorder” and “MicroRNAs”. The literature search was conducted on 12 March 2025, without date restriction. The complete database-specific search strategies and query strings are provided in Supplementary Table S1 to ensure transparency and reproducibility. We also cross-referenced relevant studies cited in previous reviews to ensure the inclusion of all eligible publications.

### 2.2. Study Eligibility Criteria

To obtain a comprehensive compilation of miRNAs associated with ASD, specific criteria were defined to ensure only the inclusion of relevant, high-quality studies, as defined below. This strategy enabled the construction of a database that integrates gene expression and genetic variant data from case–control studies investigating human miRNAs in the context of ASD.

Eligible studies included peer-reviewed publications or dissertations published in English. The inclusion criteria were (a) children and adults with an ASD diagnosis according to the Diagnostic and Statistical Manual of Mental Disorders (DSM) or another validated instrument, including the Autism Diagnostic Observation Schedule (ADOS), the Autism Diagnostic Interview-Revised (ADI-R), and the Childhood Autism Rating Scale (CARS); (b) studies investigating miRNA profiling, gene expression, or genetic variants; (c) data generated using validated experimental methodologies; and (d) studies reporting statistically significant differences in miRNA expression or genetic variants between individuals with ASD and neurotypical controls. Participants with comorbidities were not excluded.

The following studies were excluded: (a) literature reviews; (b) book sections; (c) letters to the editor, (d) conference summaries, (e) hypothesis papers, (f) studies without case-control analysis, (g) duplicated data, and (h) non-human studies.

### 2.3. Data Extraction and Standardization

All eligible studies were manually reviewed for data extraction and categorized into two groups: ‘Expression studies’ and ‘Genetic and Other studies’.

For each publication included in the ‘Expression studies’ group, data were extracted into a standardized template including: first author, year of publication, tissue type(s), sample size(s), diagnostic criteria, assay methodology, country of origin, and lists of upregulated and downregulated miRNAs with statistically significant expression changes. Two publications used computational approaches based on expression data from case–control studies to identify ASD-associated miRNAs but did not report differential expression analyses. As these studies did not provide lists of differentially expressed miRNAs, they were curated separately and classified as ‘Bioinformatics’ within the ‘Genetic and Other Studies’ group.

Studies investigating genetic variants in miRNA genes were also included within the ‘Genetic and Other studies’ group. For each study, data were extracted into a separate standardized template including the first author, year of publication, affected miRNA gene(s), sample size(s), diagnostic criteria, assay methodology, country of origin, and type of genetic variant, namely single nucleotide variants (SNVs), copy number variants (CNVs), and single nucleotide polymorphisms (SNPs).

To ensure consistency and facilitate comparisons across studies published over nearly two decades, all reported miRNA identifiers were manually harmonized to the current miRBase v22 [12] nomenclature, resolving historical naming inconsistencies across studies. Only statistically significant results reported in the original publications were included in the database. When inconsistencies in the direction of miRNA dysregulation were observed across studies, all reported findings were retained together with information on tissue type and experimental methodology, allowing users to assess reproducibility and potential sources of heterogeneity.

### 2.4. External Resources

In addition to the manually curated data obtained from the literature, *miR4ASD* integrates information from external resources to provide functional context for ASD-associated miRNAs.

Experimentally validated miRNA–target gene interactions (MTIs) were retrieved from miRTarBase v10.0 [13]. For each interaction, information on the miRNA, target gene, supporting experimental methods, and PubMed reference was integrated into the database. Additionally, information on genes implicated in ASD was obtained from SFARI Gene [14], allowing for the identification of target genes according to the strength of evidence supporting their involvement in ASD risk.

Functional enrichment analysis was implemented using the g:Profiler REST API [15], enabling users to perform enrichment analyses based on selected miRNAs and/or target genes directly within *miR4ASD*.

### 2.5. Database Implementation

The *miR4ASD* database is hosted on GitHub (https://mir4ASD.github.io/miR4ASD) under a Creative Commons Attribution-ShareAlike 4.0 International License. It was implemented as a static web application, developed using standard web technologies: HyperText Markup Language (HTML) for structure, Cascading Style Sheets (CSS) for styling, and JavaScript for interactive functionality. Manually curated data were stored in JavaScript Object Notation (JSON) files that are dynamically loaded by the JavaScript table code. Interactive tables with pagination, search and filtering functionalities were implemented using the DataTables JavaScript library (https://datatables.net/, version 2.0.8).

The *miR4ASD* database is intended to be regularly updated to incorporate newly published studies and future miRBase releases, ensuring its continued relevance as a resource for ASD research. All code is freely available on a GitHub repository (https://github.com/miR4ASD/miR4ASD/).

### 2.6. Database Structure: Filtering and Searching

The user interface displays data in six main categories: ‘About *miR4ASD*’, ‘Expression Studies’, ‘Genetic & Other Studies’, ‘Target Genes’, ‘Functional Enrichment’, and ‘Help’, organized in a tabbed layout.

The ‘About *miR4ASD*’ section provides an overview of the resource, including its objectives, curation methodology, and general statistics summarizing the curated literature. The “Expression Studies” section contains publications reporting differential miRNA expression in ASD case–control studies, whereas the ‘Genetic & Other Studies’ section includes investigations reporting genetic variants in miRNA genes, as well as computational studies that identified ASD-associated miRNAs using expression-based approaches without performing conventional differential expression analyses. Both sections are presented as interactive tables, in which each entry represents a single miRNA, together with the curated evidence supporting its association with ASD. Expanding an entry reveals the manually curated detailed information extracted from the original studies and includes direct links to the supporting publications. Hairpin and mature miRNA identifiers were cross-referenced with the human miRBase General Feature Format (GFF) annotation file and hyperlinked to their corresponding miRBase v22 entries (https://www.mirbase.org/download/hsa.gff3, version date 5 March 2018), providing access to standardized sequence and annotation information.

The ‘Target Genes’ section contains experimentally validated MTIs obtained from miRTarBase. Users can explore the target genes of each selected miRNA, together with information on the experimental methods supporting each interaction and the strength of evidence implicating these target genes in ASD risk. The ‘Functional Enrichment’ section allows users to perform a functional enrichment analysis of target genes associated with selected miRNAs using g:Profiler. Finally, the ‘Help’ section provides detailed guidance on the database content, functionality, and usage, including descriptions of the information presented in each section.

The platform allows users to search, filter, and download data to ensure accessibility and reproducibility. Section-specific filters enable users to explore the data according to relevant criteria. For instance, in the ‘Expression Studies’ and ‘Genetic & Other Studies’ sections, users can filter miRNAs by name, expression change (upregulated or downregulated), biological sample type, genetic variant type, overall evidence, methodology, diagnostic tools, and number of supporting studies. Users can also select specific miRNAs identified in expression, genetic, or bioinformatics studies and directly explore their experimentally validated target genes. In the ‘Target genes’ section, users can filter MTIs by target gene name, ASD susceptibility (SFARI category), experimental support type, and methodology. Finally, users can perform functional enrichment analysis of selected targets directly within *miR4ASD*.

## 3. Results

### 3.1. Summary of Eligible Studies

After screening records identified across the PubMed, Web of Science, and Scopus databases, a total of 59 studies comparing individuals with ASD and neurotypical controls were included in *miR4ASD* (Supplementary Table S2). The studies were published between 2008 and 2025 and conducted across multiple countries, including Bulgaria, China, Egypt, France, Iran, Italy, Japan, Qatar, the United Kingdom (UK), and the United States of America (USA), with the USA contributing the highest number of publications.

Across all studies, ASD diagnoses were generally established using validated diagnostic instruments and/or criteria from the DSM, namely the 5th Edition (DSM-5), the 4th Edition Text Revision (DSM-IV-TR), or the 4th Edition (DSM-IV). Frequently used assessment tools included the ADI-R, the ADOS, and the CARS, either alone or in combination with the DSM criteria. Three studies did not specify the diagnostic method, but ASD samples were obtained from brain tissue banks or publicly available ASD databases.

Of the 59 studies included, 50 were gene expression or miRNA profiling studies, 7 were genetic analyses, and 2 were bioinformatics studies (Supplementary Table S2). This distribution highlights that the current evidence on miRNAs in ASD is dominated by expression-based studies, whereas comparatively few investigations have explored genetic variation in miRNA genes.

Among the expression studies, tissue sources were diverse, with the majority analyzing peripheral blood (*n* = 29), including serum, plasma, blood cells, exosomes, and whole blood. Other biospecimens included post-mortem brain tissue (*n* = 6), saliva (*n* = 6), lymphoblastoid cell lines (LCLs, *n* = 6), neural stem cells (NSC, *n* = 1), olfactory mucosal stem cells (OMSCs, *n* = 1), pineal glands (*n* = 1), and umbilical cord blood (*n* = 1), which reflects the methodological diversity of the field. In addition, two bioinformatics studies identified ASD-associated miRNAs using computational approaches based on expression data. These studies employed either a knowledge-guided bioinformatics framework for miRNA biomarker prioritization [16] or a saliva-based RNA classification algorithm for ASD diagnosis [17]. The genetic studies comprised analyses of CNVs (*n* = 3), SNVs (*n* = 2), and SNPs (*n* = 2).

### 3.2. Differentially Expressed miRNAs

In the 50 miRNA expression studies, a total of 384 mature miRNAs were reported as differentially expressed in individuals with ASD compared with neurotypical controls. Of these, 64 miRNAs were reported in three or more studies, and 134 miRNAs were identified in at least two independent studies. The subset of mature miRNAs identified in at least three independent studies is provided in Supplementary Table S3, organized by tissue type, to highlight both reproducible and tissue-specific patterns of dysregulation.

**Figure 1:**
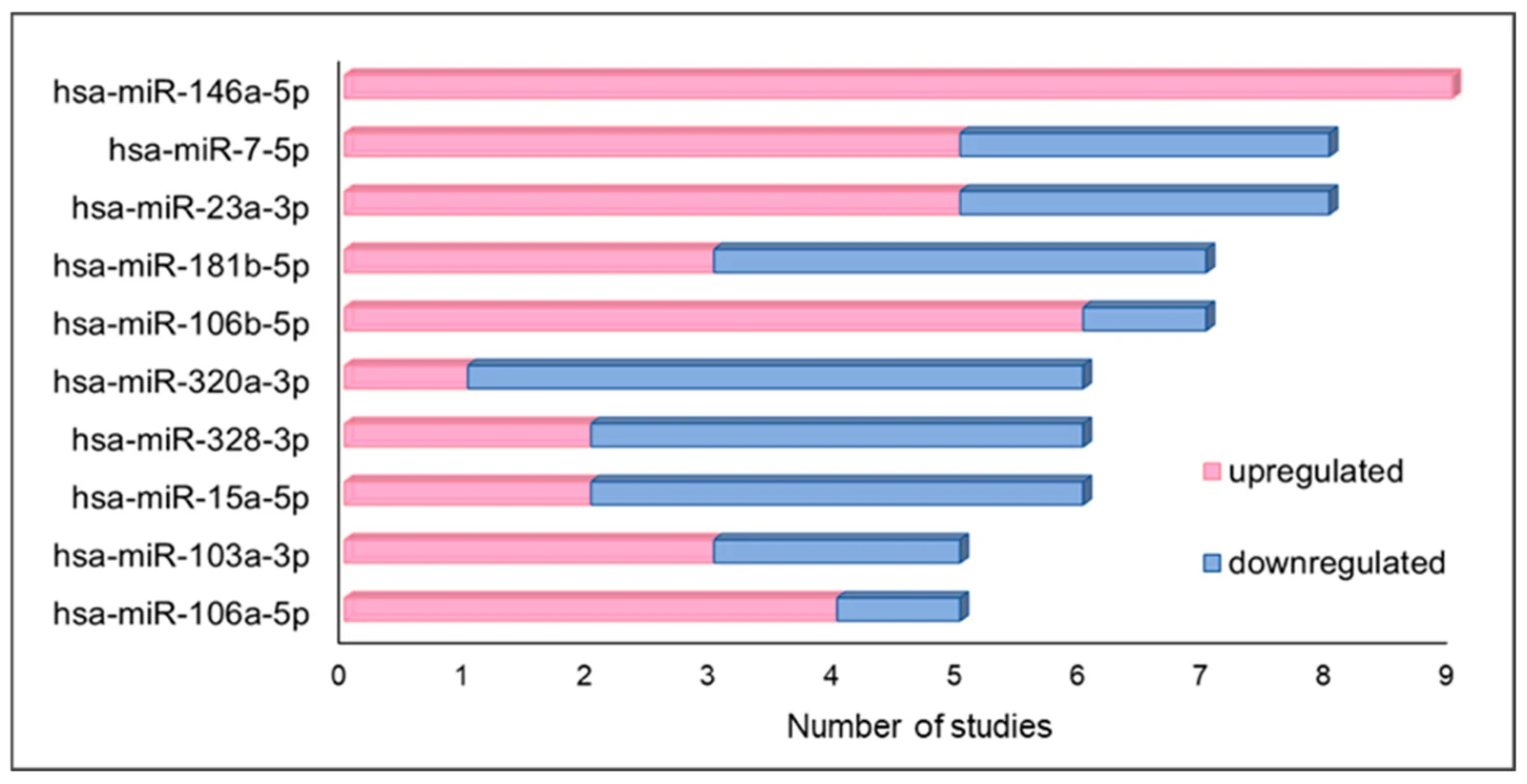
Top 10 mature miRNAs most frequently reported in ASD expression studies. Mature miRNAs are ranked according to the number of independent publications reporting statistically significant differential expression in ASD case–control studies.

Several miRNAs demonstrated consistent dysregulation across multiple tissue types, whereas others displayed tissue-specific expression patterns. Among the most frequently replicated findings, hsa-miR-146a-5p stood out as the most consistently reported, identified as upregulated in nine independent studies across peripheral blood-derived samples, saliva, post-mortem brain tissue, LCLs, and OMSCs [18,19,20,21,22,23,24,25,26] (Figure 1).

Other miRNAs consistently reported as upregulated in at least four independent studies spanning multiple tissue types included hsa-miR-107, hsa-miR-142-3p, hsa-miR-155-5p, hsa-miR-191-5p, and hsa-miR-223-3p (Supplementary Table S3) [6,19,23,26,27,28,29,30,31,32,33]. In contrast, hsa-miR-92a-3p was the only miRNA consistently reported as downregulated in four independent studies, all of which analyzed peripheral blood-derived samples or LCLs [18,26,28,34].

In addition to these reproducible trends, 25 miRNAs exhibited inconsistent dysregulation, being reported as either upregulated or downregulated in four or more studies. Notably, hsa-miR-23a-3p and hsa-miR-7-5p were each reported as upregulated in five studies but downregulated in three studies across multiple tissue types [6,18,19,26,27,28,31,35,36,37,38,39] (Figure 1). Similarly, hsa-miR-106b-5p was reported as upregulated in six studies involving peripheral blood-derived samples, post-mortem brain tissue, and LCLs [24,29,35,36,40], but downregulated in a single study of LCLs [27]. Conversely, hsa-miR-320a-3p showed the opposite trend, being reported as downregulated in five studies involving peripheral blood-derived samples or LCLs [18,40,41,42,43], but upregulated in one study of post-mortem brain tissue [36] (Figure 1).

Overall, the curated studies revealed both reproducible and conflicting patterns of miRNA dysregulation across tissues and independent cohorts.

### 3.3. Genetic Variants and Other Evidence for miRNA-ASD Associations

In addition to differential expression studies, *miR4ASD* also incorporates findings from nine studies that examined miRNA associations with ASD through either genetic or bioinformatics analyses [16,17,44,45,46,47,48,49,50]. Across these studies, 93 miRNA genes were associated with ASD in at least one publication. Of these, eight miRNA genes were independently identified in two genetic or bioinformatics studies (Table 1).

**Figure 2:**
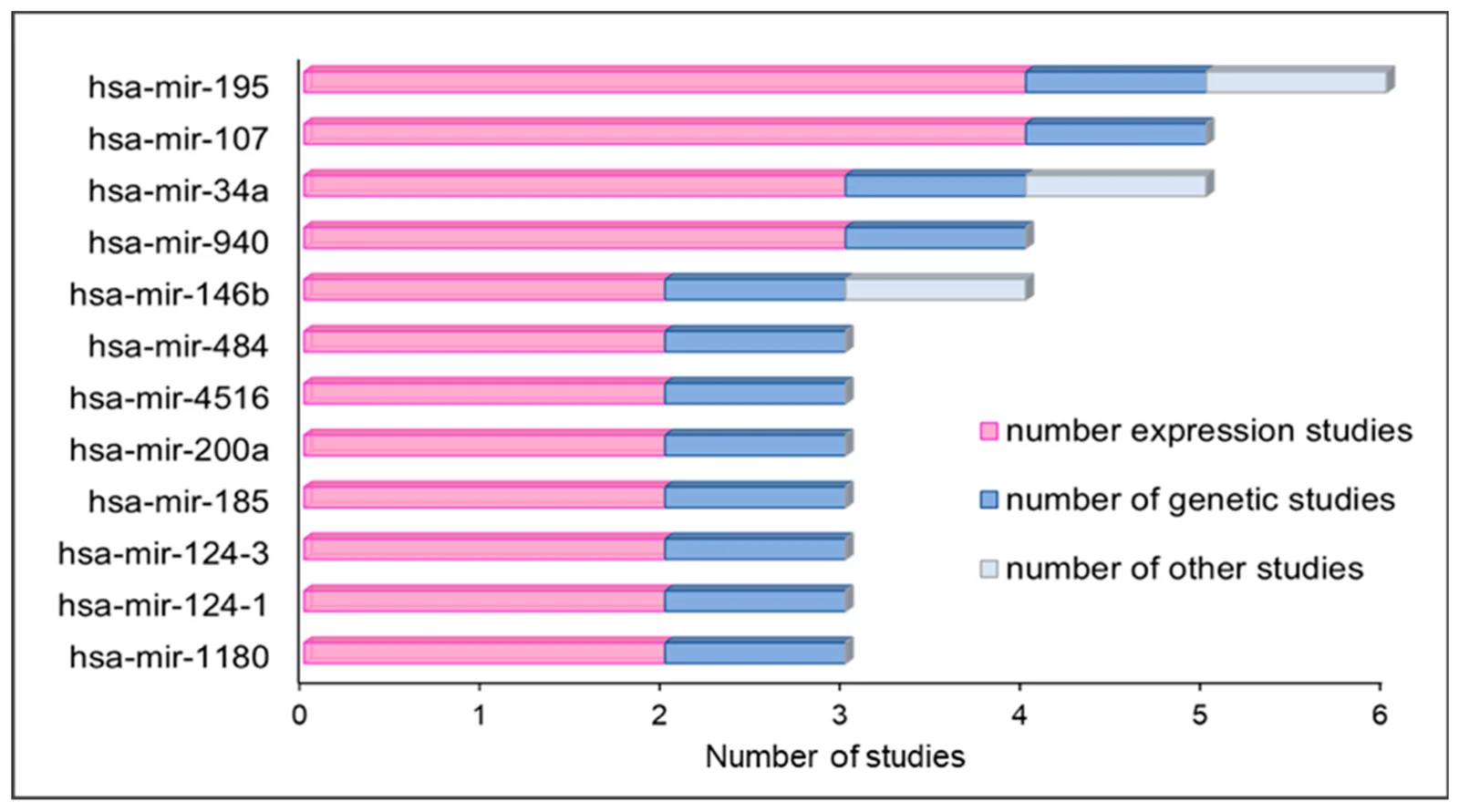
Twelve miRNA genes implicated in ASD, supported by complementary evidence curated in the *miR4ASD* database. The figure highlights the miRNA genes identified across multiple study types, including differential expression studies, genetic analyses, and other studies. The “other studies” category refers to computational studies that identified ASD-associated miRNAs using expression-based bioinformatics analyses.

Overall, 29 unique miRNA genes were supported by evidence from at least two independent studies, either through multiple genetic or bioinformatics studies or through one such study together with replication in differential expression studies (Table 1). Of these, 24 miRNA genes were supported by differential expression in at least two independent expression studies, providing complementary evidence across different study designs (Table 1). Notably, twelve miRNA genes were independently identified in three or more studies spanning differential expression, genetic, and bioinformatics approaches, representing the strongest convergent evidence curated in *miR4ASD* (Figure 2, Table 1).

### 3.4. Target Genes and Functional Enrichment

To provide additional functional information for the miRNAs included in *miR4ASD*, miRNA–target interaction data were integrated from miRTarBase v10.0. Overall, 17,150 MTIs were identified for the 466 unique mature miRNAs implicated in ASD, comprising 2995 unique target genes. Of these, 846 genes were classified as ASD risk genes according to SFARI Gene, with 143 classified as Category 1, 205 as Category 2, 423 as Category 3, and 75 as Syndromic. Among the 466 ASD-associated miRNAs, 332 target at least one gene classified as high confidence (SFARI category 1).

Functional enrichment analysis, implemented using g:Profiler REST API, can be performed based on a user-defined selection of miRNAs and/or specific MTIs, using the corresponding target genes for the analysis. Enrichment results may include Gene Ontology (GO, Molecular Function, Biological Process, and Cellular Component), KEGG pathways, Reactome pathways, WikiPathways, and Human Phenotype Ontology (HP) terms. Users can also select among the multiple testing correction methods provided by g:Profiler, including g:SCS algorithm (default), Bonferroni correction, and the Benjamini–Hochberg false discovery rate (FDR), and define the significance threshold applied to the enrichment results.

Together, these functionalities provide additional functional context for the miRNAs associated with ASD in *miR4ASD* and facilitate the exploration of biological processes and pathways associated with their target genes.

### 3.5. Data Visualization

**Figure 3:**
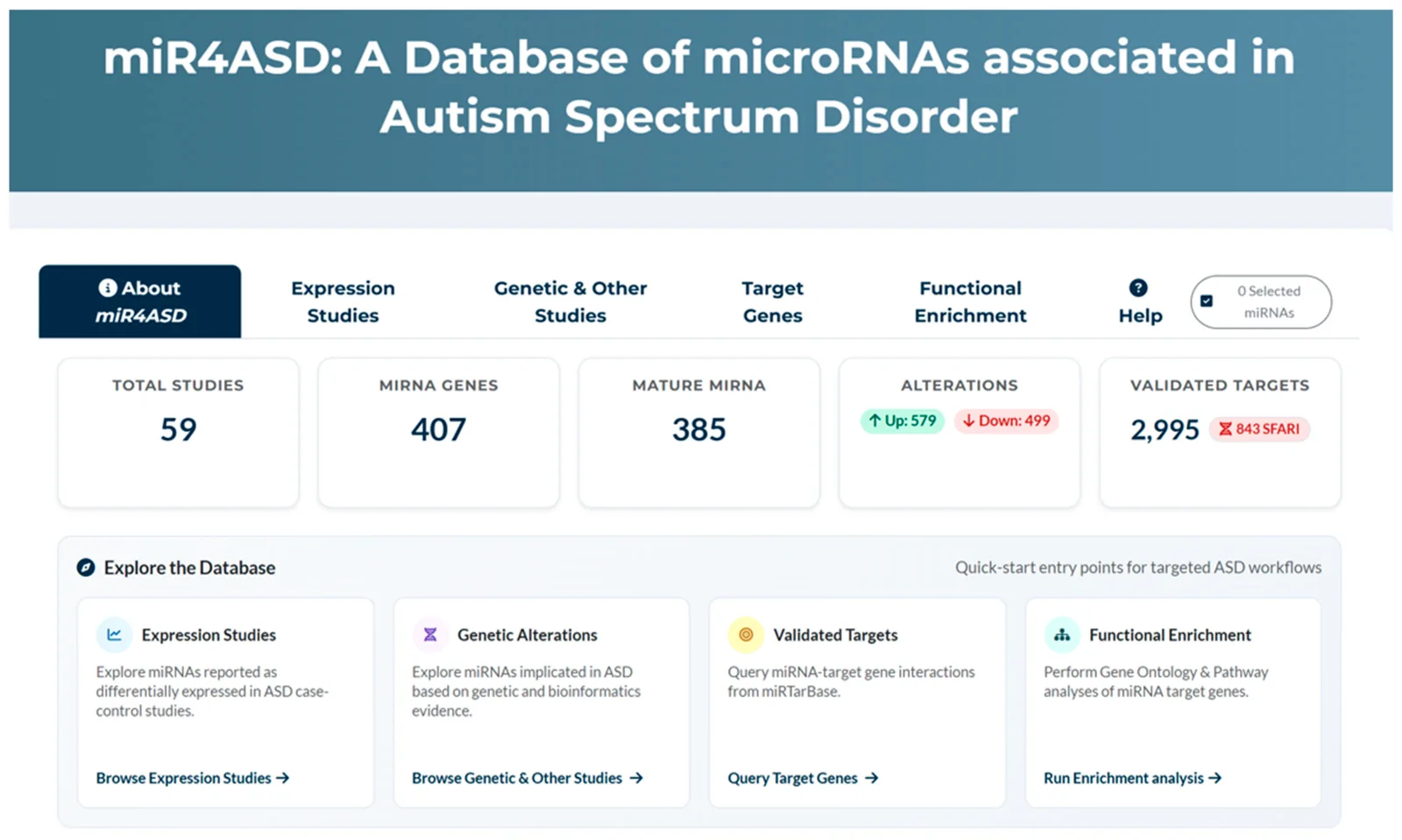
*miR4ASD* homepage (https://mir4asd.github.io/miR4ASD/#about). Overview of the database including its objectives, organization, and summary statistics. Navigation is organized into six main sections: ‘About *miR4ASD*’, ‘Expression Studies’, ‘Genetic & Other Studies’, ‘Target Genes’, ‘Functional Enrichment’ and ‘Help’.

The *miR4ASD* database is publicly available through a web interface designed to facilitate intuitive exploration of curated miRNA data. The homepage provides an overview of the resource, including its objectives, summary statistics, and access to the six main database sections: ‘About *miR4ASD*’, ‘Expression Studies’, ‘Genetic & Other Studies’, ‘Target Genes’, ‘Functional Enrichment’ and ‘Help’ (Figure 3).

**Figure 4:**
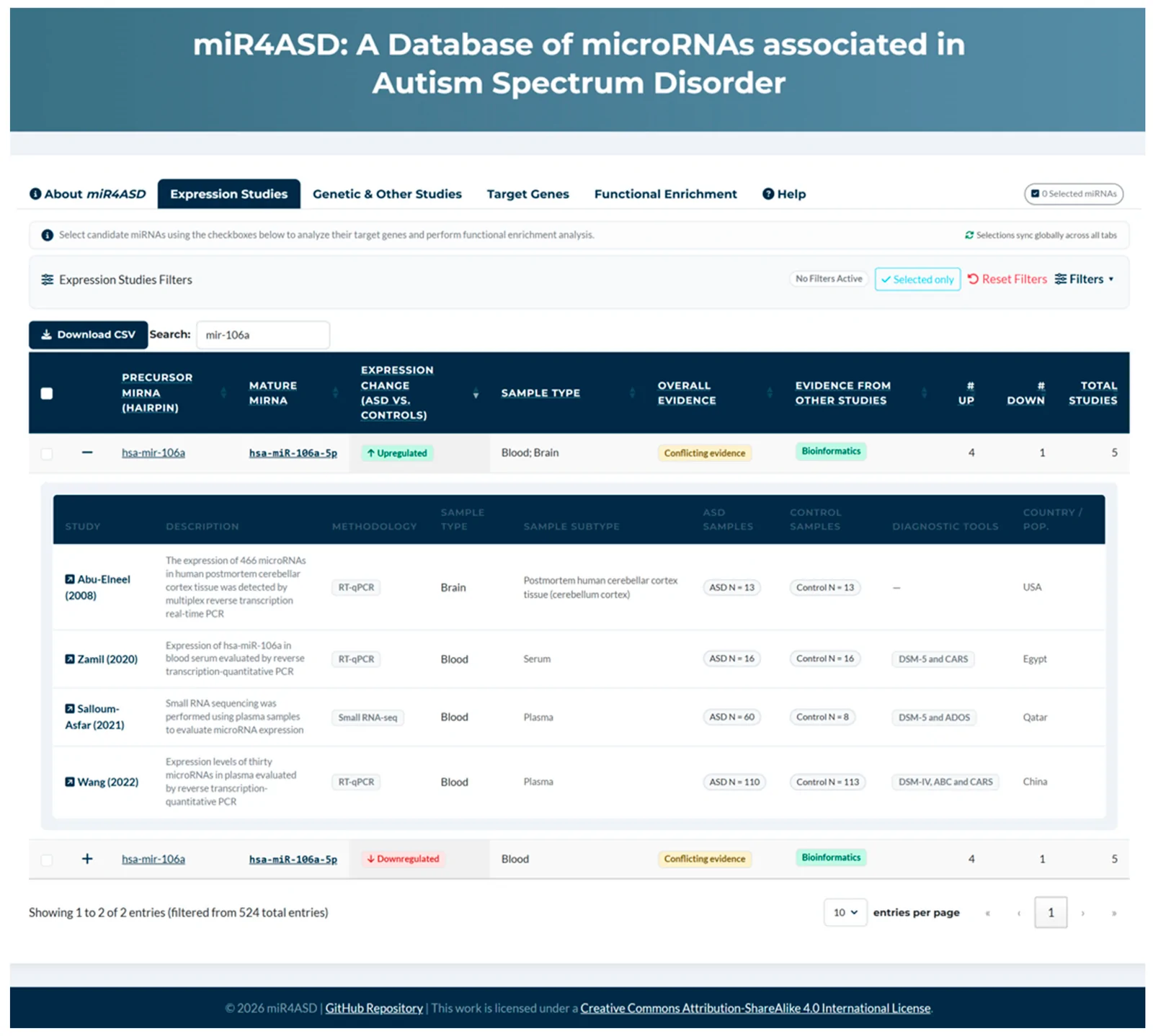
Search and filtering functionalities of the ‘Expression Studies’ section in *miR4ASD*. Each entry displays the precursor (hairpin) and mature miRNA identifiers (standardized according to miRBase v22), expression profile, and summary of the supporting evidence, allowing users to efficiently search, sort, and filter the curated dataset (https://mir4asd.github.io/miR4ASD/#expression). The detailed information from original studies is accessible by clicking the “+” button associated with each miRNA entry. The figure shows an example of the results obtained from a specific search for the mir-106a gene.

The ‘Expression Studies’ section provides access to all curated miRNAs reported as differentially expressed in ASD case–control studies (Figure 4). Each row corresponds to a single miRNA (including the mature and the corresponding precursor identifier), and contains information on expression profile, sample type, supporting evidence and the number of studies reporting increased or decreased expression. The detailed information from original studies is accessible by clicking the “+” button associated with each miRNA entry.

Users can search, filter, and sort the table according to individual columns, facilitating the identification of specific miRNAs or expression patterns. Expanding an entry displays the complete manually curated evidence, including tissue type, sample size, diagnostic criteria, country of origin, experimental methodology, expression direction, and direct links to the original publications. Hairpin and mature miRNA identifiers are hyperlinked to their corresponding miRBase entries, providing immediate access to standardized nomenclature and sequence annotation. Figure 4 shows an example of a search for the hsa-mir-106a gene, which was reported as upregulated in four studies and downregulated in one study.

**Figure 5:**
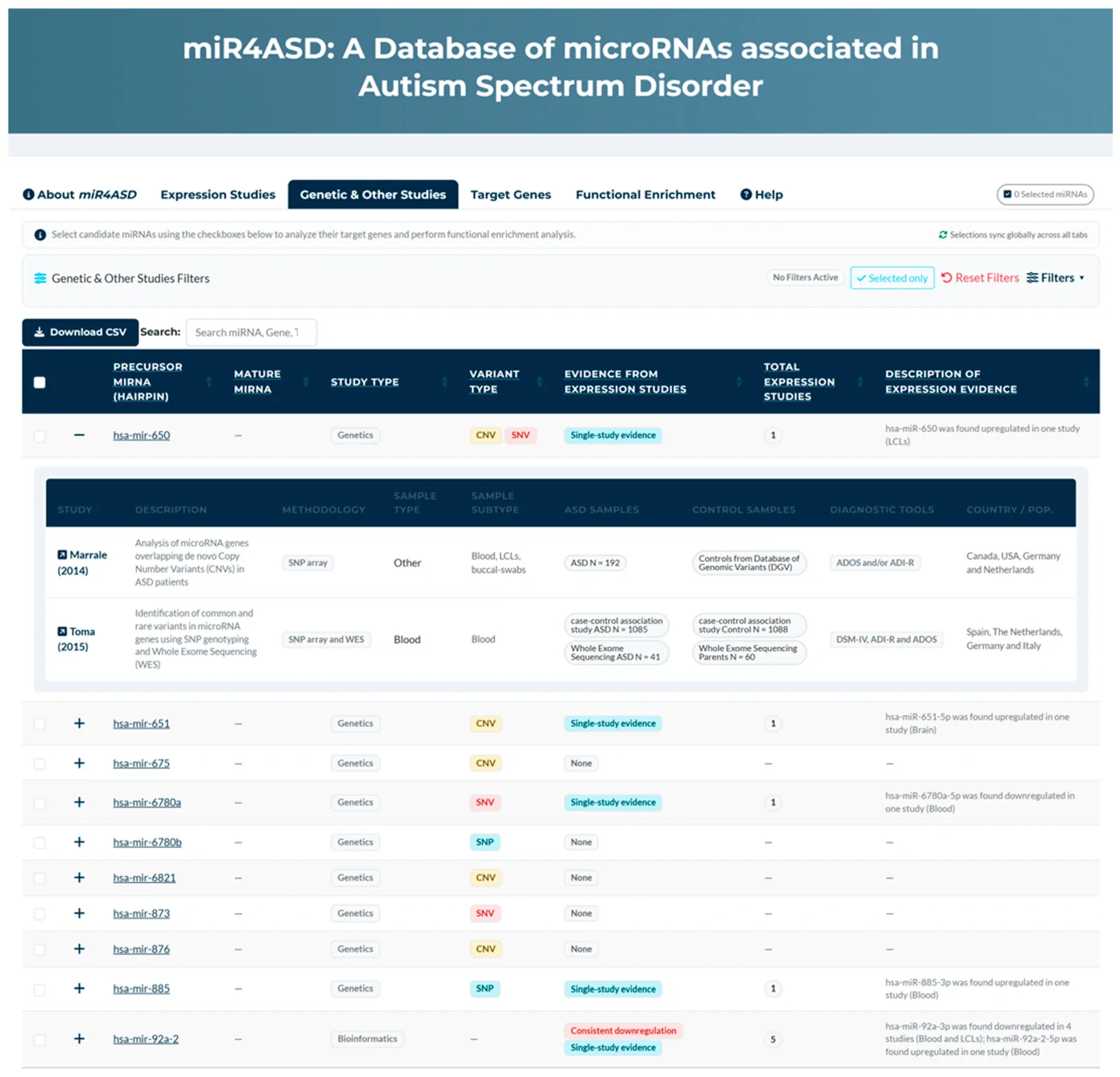
Search and filtering functionalities of the ‘Genetic & Other Studies’ section in *miR4ASD*. Each entry displays the precursor (hairpin) miRNA identifier, study type, reported variant type, and information on additional evidence from the expression studies. The detailed information from original studies is accessible by clicking the “+” button associated with each miRNA entry (https://mir4asd.github.io/miR4ASD/#genetic).

The ‘Genetic & Other Studies’ section provides access to miRNA genes associated with ASD through genetic analyses or expression-based bioinformatics approaches (Figure 5).

Each row corresponds to a single miRNA (including the mature and the corresponding precursor identifier) and contains information on study type, variant type, evidence from expression studies, and the number of studies reporting altered miRNA expression. Similar to the ‘Expression Studies’ section, users can search, filter, and sort the table according to individual columns. Expanding an entry reveals the complete manually curated information extracted from the original publications, together with direct links to the corresponding articles.

The ‘Target Genes’ section provides access to miRNA–target interactions for the miRNAs included in *miR4ASD.* Each row corresponds to a single miRNA–target interaction and contains information on the mature and precursor miRNA identifiers, target gene, experimental support type, experimental methodologies, PubMed reference, and when available, the SFARI Gene score and corresponding evidence category.

The ‘Functional Enrichment’ section allows users to perform a functional enrichment analysis of target genes associated with selected miRNAs and/or specific miRNA-target interactions. Users can select the enrichment data sources, multiple testing correction method, and significance threshold. Results are displayed in an interactive table containing the enriched terms, corresponding data source, statistical significance, and associated target genes.

The ‘Help’ section provides detailed guidance on the content, functionality, and use of *miR4ASD*. It includes descriptions of the information presented in each database section, together with explanations of the experimental methodologies, clinical diagnostic tools, and external resources used in the database.

This interface was designed to facilitate the identification and exploration of ASD-associated miRNAs across independent studies, enabling users to compare findings from different tissues, methodologies, populations, and study designs. By integrating these data into a standardized and searchable resource, *miR4ASD* supports transparency, reproducibility, and accessibility for the ASD research community.

## 4. Discussion

*miR4ASD* represents an important step toward integrating dispersed knowledge, concerning the involvement of miRNA in ASD etiology and pathophysiology, into a user-friendly and open-source format. By consolidating data from 59 studies, including expression profiling, genetic variants, and bioinformatics analyses, this resource provides an integrated platform that overcomes the fragmentation of the current literature. The database highlights both convergence and variability in the reported findings. While hundreds of miRNAs have been associated to ASD, only a smaller subset shows reproducible associations across independent cohorts, biological tissues, and study designs. By structuring these data and standardizing nomenclature, *miR4ASD* offers an accessible and evolving tool to guide biomarker discovery, mechanistic functional studies, and translational research in ASD.

The expression studies included in *miR4ASD* illustrate both the strengths and limitations of the current evidence on miRNA dysregulation in ASD. Several miRNAs emerged as reproducible candidates. For example, hsa-miR-146a-5p was consistently upregulated across nine independent studies involving blood, saliva, post-mortem brain tissue, LCLs, and OMSCs [18,19,20,21,22,23,24,25,26]. Notably, this miRNA was also identified as one of the informative RNA biomarkers incorporated into a quantitative salivary RNA-based classifier capable of distinguishing children with ASD from those with developmental delay or typical development [17].

The database also highlights miRNAs reported in multiple studies but with inconsistent directionality. Such inconsistencies may reflect true biological heterogeneity, such as tissue-specific regulation or biological diversity among cohorts, but may also arise from technical variability in experimental design and statistical analysis. A representative example is hsa-miR-320a-3p, which was reported as downregulated in most studies using peripheral blood-derived samples or LCLs [18,40,41,42,43], whereas one study reported increased expression in post-mortem brain tissue [36]. This apparent discrepancy likely reflects the influence of tissue-specific miRNA regulation, highlighting the importance of considering the biological source when interpreting expression data. Nevertheless, the recurrent identification of hsa-miR-320a-3p and other miRNAs showing inconsistent directionality across independent cohorts and biological specimens supports their biological relevance and warrants further investigation. By systematically integrating these findings within a standardized framework, *miR4ASD* enables researchers to identify consistently replicated miRNAs, recognize conflicting evidence, and prioritize candidates for future functional validation.

Beyond expression profiling, *miR4ASD* also integrates evidence from genetic studies and bioinformatics analyses, enabling complementary lines of evidence to be explored within a single resource. For example, hsa-mir-1914 and hsa-mir-650 were independently implicated by both SNV [46] and CNV studies [44,45], suggesting that these miRNA genes may be affected through distinct classes of genetic variation. While functional evidence for these candidates is currently lacking, their recurrent identification across independent genetic approaches highlights them as promising targets for future experimental investigation. Despite these encouraging findings, the curated literature highlights that genetic studies focusing on miRNA genes in ASD remain scarce, generally involving relatively small cohorts and showing limited replication across independent datasets [46,50]. This gap limits our understanding of how genetic variation in miRNA genes contributes to ASD susceptibility and phenotypic heterogeneity. These observations reinforce the value of *miR4ASD*, which provides multiple lines of evidence within a single resource and helps researchers prioritize the most promising miRNA candidates.

Importantly, the integration of cumulative evidence from the differential expression, genetic, and bioinformatics studies included in *miR4ASD* enabled the identification of twelve miRNA genes consistently implicated in ASD across three or more independent studies. This convergence of evidence strengthens the support for these candidate miRNAs, highlighting the utility of *miR4ASD* as an integrative database for generating hypotheses and guiding future functional and translational research in ASD. Moreover, this resource also integrates complementary information from external resources, namely miRNA–target interactions and SFARI Gene scores, while also providing functional enrichment analysis. This additional layer of information extends the utility of the database by allowing users to explore the potential functional context of miRNAs implicated in ASD. The ability to perform specific enrichment analyses based on selected miRNAs further facilitates the investigation of biological processes and pathways associated with their target genes.

The current version of *miR4ASD* represents an initial step toward integrating diverse evidence on miRNAs in ASD. At this stage, the database primarily includes case–control studies, providing a solid foundation for comparative and exploratory analyses. However, research on miRNAs in ASD is still an emerging field, and *miR4ASD* was designed as a dynamic resource that can be regularly updated and evolve alongside the growing body of evidence. Future updates will incorporate newly available data from external resources, namely miRBase, miRTarBase, and SFARI Gene, and may broaden its scope to include studies comparing ASD with other neurodevelopmental disorders and evidence linking miRNA dysregulation to specific ASD phenotypes. Together, these additions may provide a more comprehensive understanding of the functional and phenotypic relevance of miRNA dysregulation in ASD.

Overall, *miR4ASD* integrates multiple lines of evidence supporting specific miRNAs as promising candidates for further investigation in ASD. By providing a standardized and manually curated platform, it facilitates the comparison of findings across studies, candidate prioritization, and reproducible research. Importantly, its dynamic nature enables the incorporation of new evidence as the field evolves, overcoming a key limitation of conventional literature reviews and providing a foundation for future studies of miRNA function, biomarker discovery, and a deeper understanding of the molecular mechanisms underlying ASD.

## Figures and Tables

**Table 1:** List of miRNA genes associated with ASD through genetic variant analyses or bioinformatics studies. This table includes miRNAs reported in at least two such studies, as well as miRNAs identified in one of these study types but also replicated in studies reporting differentially expressed miRNAs. Each entry specifies the miRNA gene, the study type (Genetics or Bioinformatics), the reported alteration, and the supporting reference. Precursor (hairpin) and mature miRNA names according to miRBase v22.

Precursor miRNA	Mature miRNA	Study Type	Alteration	Study	Expression Studies ^a^
hsa-mir-34a	hsa-miR-34a-3p hsa-miR-34a-5p	Genetics; Bioinformatics	CNV; miRNA expression	Vaishnavi (2013); Shen (2016) [16,44]	3 studies
hsa-mir-96	hsa-miR-96-5p	Bioinformatics	miRNA expression	Shen (2016) [16]	3 studies
hsa-mir-106a	miR-106a-5p	Bioinformatics	miRNA expression	Hicks (2018) [17]	5 studies
hsa-mir-106b	miR-106b-5p	Bioinformatics	miRNA expression	Shen (2016) [16]	7 studies
hsa-mir-107		Genetics	CNV	Qiu (2023) [50]	4 studies
hsa-mir-146a	hsa-miR-146a-3p hsa-miR-146a-5p	Bioinformatics	miRNA expression	Hicks (2018) [17]	9 studies
hsa-mir-146b	hsa-miR-146b-3p hsa-miR-146b-5p	Genetics; Bioinformatics	SNP; miRNA expression	ASDWGPGC (2017); Hicks (2018) [17,47]	2 studies
hsa-mir-185		Genetics	CNV	Marrale (2014) [45]	2 studies
hsa-mir-186	hsa-miR-186-5p	Bioinformatics	miRNA expression	Shen (2016) [16]	2 studies
hsa-mir-195	hsa-miR-195-5p	Genetics; Bioinformatics	CNV; miRNA expression	Vaishnavi (2013); Shen (2016) [16,44]	4 studies
hsa-mir-200b		Genetics	CNV	Vaishnavi (2013); Marrale (2014) [44,45]	NR
hsa-mir-200a		Genetics	CNV	Marrale (2014) [45]	2 studies
hsa-mir-205	hsa-miR-205-5p	Bioinformatics	miRNA expression	Shen (2016) [16]	2 studies
hsa-mir-211	hsa-miR-211-5p	Bioinformatics	miRNA expression	Shen (2016) [16]	2 studies
hsa-mir-429		Genetics	CNV	Vaishnavi (2013); Marrale (2014) [44,45]	NR
hsa-mir-484		Genetics	CNV	Vaishnavi (2013) [44]	2 studies
hsa-mir-650		Genetics	CNV; SNV	Marrale (2014); Toma (2015) [45,46]	NR
hsa-mir-940		Genetics	CNV	Qiu (2023) [50]	3 studies
hsa-mir-1180		Genetics	CNV	Vaishnavi (2013) [44]	2 studies
hsa-mir-1306		Genetics	CNV	Vaishnavi (2013); Marrale (2014) [44,45]	NR
hsa-mir-1914		Genetics	CNV; SNV	Vaishnavi (2013); Toma (2015) [44,46]	NR
hsa-mir-4516		Genetics	CNV	Qiu (2023) [50]	2 studies
hsa-mir-124-1		Genetics	CNV	Vaishnavi (2013) [44]	2 studies
hsa-mir-124-3		Genetics	CNV	Vaishnavi (2013) [44]	2 studies
hsa-mir-181b-1	hsa-miR-181b-5p	Bioinformatics	miRNA expression	Shen (2016) [16]	7 studies
hsa-mir-181b-2	hsa-miR-181b-5p	Bioinformatics	miRNA expression	Shen (2016) [16]	7 studies
hsa-mir-193b	hsa-miR-193b-3p	Bioinformatics	miRNA expression	Shen (2016) [16]	2 studies
hsa-mir-486-1	hsa-miR-486-5p	Bioinformatics	miRNA expression	Shen (2016) [16]	2 studies
hsa-mir-92a-2	miR-92a-3p	Bioinformatics	miRNA expression	Hicks (2018) [17]	4 studies

**Abbreviations:** ASDWGPGC, The Autism Spectrum Disorders Working Group of The Psychiatric Genomics Consortium; CNV, copy number variant; SNV, single nucleotide variant; SNP, single nucleotide polymorphism; NR, not reported. ^a^ Number of expression studies reporting each miRNA as upregulated or downregulated.

## Data Availability

No new data were created or analyzed in this study. Data sharing is not applicable to this article.

## Supplementary Materials

The following supporting information can be downloaded at: https://www.mdpi.com/article/10.3390/cimb48101044/s1.
